# The “Why” in Mental Health, Stigma, and Addictive Behaviors: Causal Inferences in Applied Settings

**DOI:** 10.3390/ijerph20206915

**Published:** 2023-10-13

**Authors:** Iván Sánchez-Iglesias

**Affiliations:** Department of Psychobiology & Behavioral Sciences Methods, Complutense University of Madrid, 28223 Madrid, Spain; i.sanchez@psi.ucm.es

## 1. Introduction

Mental health problems, broadly understood, are highly prevalent. Depression, anxiety, or burnout in the workplace are just some of the disorders so frequent that we forget their great impact on health and the functioning of society. When it comes to severe mental illnesses, such as schizophrenia and other psychotic disorders, we may encounter stigmatization, prejudice, and discrimination against the people who suffer from them. However, stigma is also related to other populations, such as homeless people or people with intellectual disabilities. There may even be comorbidity between these conditions and mental health problems. Other factors—such as gender—seem to be related, and their study may have an impact on social and equity policies. Additionally, addictions and other addictive behaviors like compulsive gambling, video gaming, and substance abuse are highly prevalent and also carry significant stigma, becoming a public health concern.

Therefore, these three major themes are interconnected. They encompass a broad range of studies and are complex enough to have multiple theoretical models, empirical approaches, and interpretations of their results. Due to their complexity, it is particularly important to interpret the results with caution, always from what the study design and analysis allows. What are the factors that lead to these conditions? What are the effects of these conditions on other relevant health outcomes? How substantial is the impact of the studies on these phenomena on clinical, social, or psychological policies?

I want to briefly reflect on this question, the “why”, in these three health-related aspects. By doing so, I hope to contribute to a better understanding of the importance of rigorous methodology in scientific research and publication, at least, as far as causal inference is concerned. In this paper, I aim to address an issue that arises in scientific publications, that is, inadequate causal claims in inferential research. To illustrate this issue, I will use mental health, stigma, and addictive behaviors as examples from three broad fields of research. 

## 2. Methodological Considerations in Scientific Research

Research in biomedical and social sciences encompasses a vast array of human-related activities and is often published in specialized and interdisciplinary journals. As a researcher with a keen interest in psychological fields, I am acutely aware of the close relationship between psychological research and biomedical issues.

When publishing the results of research, especially in peer-reviewed scientific papers (which are allegedly the gold standard for scientific reporting), we must put effort into the methodological aspects. Methodological considerations can make research more reliable, understandable, and accessible to readers who lack specialized knowledge in statistics and research methods. Unfortunately, some researchers assume that readers possess a strong understanding of fundamental concepts in methodology and statistics, which can make research inaccessible or lead to erroneous conclusions.

In addition to the risk of misunderstandings due to inadequate methodology, researchers may also intentionally overstate their findings or provide misleading information in their rush to publish, especially in competitive academic environments [1]. This leads to inaccurate conclusions and is a threat to scientific research credibility [2]. These questionable practices extend to all areas of research and at all levels of the research career [3]. However, I believe that inadequate reporting is typically not the result of intentional misrepresentation but a result of researchers taking certain aspects of their work for granted or being unaware of reporting guidelines. Many researchers are currently studying various methodological issues, including the so-called replication crisis [4], which has given rise to studies focused on meta-science. One of the areas where errors (whether intentional or unintentional) can occur in the communication of results is in the interpretation of empirical findings. Specifically, in this paper, I aim to focus on the interpretation of causal relationships between variables.

## 3. Balancing External and Internal Validity in Research Design: The Role of Sampling and Assignment in Causal Inference

As previously mentioned, the methodological aspects of studies concerning mental health, stigma, and addictive behaviors should be carefully considered. On the one hand, it is important to take into account the internal validity of a study. Experiments (i.e., studies designed based on experimental methodology) provide a higher guarantee that the observed changes in the dependent variable of interest are due to the changes introduced (through experimental manipulation) in the independent variable(s). In other words, to overcome the presence of confounding variables, which are factors that could potentially affect the outcome, researchers rely on an experimental design [5], often referred to as a randomized controlled trial (or simply a clinical trial). Correlational (i.e., non-experimental) studies, also referred to as observational studies, exhibit limited internal validity as they do not allow for random assignment, which indirectly controls for unaccounted extraneous variables in the study’s design. Nonetheless, correlational studies are also useful for causal inference, notwithstanding their constraints. Notably, epidemiological investigations have played a pivotal role in shaping numerous public health policies. While correlations themselves cannot establish causation, they can indicate potential causes and serve as a catalyst for formulating hypotheses for subsequent research. These studies assume a crucial role in establishing causal links, particularly in cumulative research [6].

Another characteristic of a study that impacts the appropriateness of causal interpretations is external validity. This effect is more subtle or indirect compared with the internal validity discussed earlier. External validity in a study is achieved through a thoughtful sampling of the population and, if possible, by using random selection. If a study does not have a representative sample of the population, its results cannot be generalized without risk. As a result, causal conclusions can be erroneous if they refer to the population when the sample does not resemble it. This can occur even when experimental methodology has been used, and there are assurances that an observed effect on a dependent variable is due to changes introduced in an independent variable [7].

**Figure 1:**
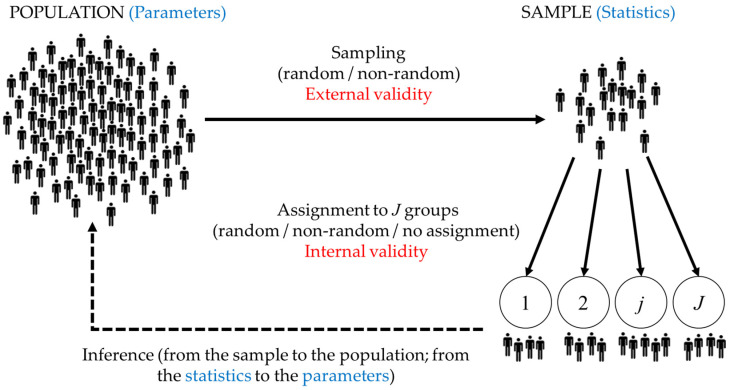
A variation of a common visual representation of the inference process that may lead to causal inference. Both sampling and assignment can be either random or non-random. However, sampling refers to the process of selecting participants from the population to be included in the study, which affects the representativeness of the sample and, therefore, its external validity. On the other hand, the assignment method (if applicable, as single-group designs do not involve assignment) is related to the guarantees of internal validity and causal inference, that is, the degree to which we can attribute changes in the dependent variable to changes in the independent variable. Inference is the process of drawing conclusions or making predictions about a population based on information obtained from a sample. It involves generalizing the sample statistics to estimate the corresponding population parameters. Inference can also involve testing hypotheses about the population parameters using the sample data. Therefore, inference involves moving from the sample to the population and from the statistics to the parameters.

Figure 1 illustrates a fundamental framework for comprehending the scientific process, particularly concerning the hypothetic-deductive method and the inferences that empirical science can draw. This visual representation might provoke a sense of frustration in the reader, as it represents basic concepts that science students are expected to grasp. Nevertheless, numerous studies have found that these essential concepts are either unknown, forgotten, or disregarded by authors and editors. In applied areas where experimentation is not feasible or ethical, correlational studies are used to accumulate scientific knowledge from evidence. However, inadequate causal inferences from correlational data appear in various fields, such as nursing [8], epidemiology [9], and nutrition [10]; in substance abuse treatment [11] and antidepressant treatments [7]; or in the effect of cannabis on the development of psychosis [12]. In these studies, authors often publish conclusions using causal language in the absence of experimental methodology. This can occur in the paper’s title, abstract, conclusions, or in multiple sections simultaneously. Regardless, such statements that lack supporting data can lead the reader to incorrect conclusions. In extreme cases, these inappropriate conclusions can prompt public or private decision makers to allocate resources to programs that may not prove effective. Hence, it becomes crucial to emphasize these concepts, if only by highlighting the applied areas where incorrect causal inferences can yield adverse consequences in clinical practice and public health policies [13,14].

## 4. Mental Health, Stigma, and Addictive Behaviors

Mental health issues are more common in society than one might think. For example, around 280 million people worldwide suffer from depression [15], and it is a health problem on the rise [16]. It is a common disorder (affecting approximately 3.8% of the population), and when it becomes recurrent (which is the case in many instances [17]), it can be a severe health problem [15]. In addition to issues like anhedonia, lack of mood, and energy, depression is associated with deaths by suicide [15,18]. Another widespread mental health issue in society is related to the work environment. The burnout syndrome is related to prolonged exposure to stressors, and it has traditionally been characterized by emotional exhaustion, depersonalization, and low personal accomplishment [19], although models with a different number of factors have also been explored [20]. Factors such as job changes, stress, caregiver burden, unrealistic expectations, and personal relationships have been studied as possible causes of the syndrome [21], as well as personality factors [22]. None of these potential causal factors can be subjected to experimental testing. However, in this area, experimental and quasi-experimental designs have been used to test the effectiveness of certain interventions against burnout [23].

Mental health disorders can also refer to other more severe conditions, such as schizophrenia or psychosis. In the study of the possible causes of psychosis, the debate about the triggering effect of substances like cannabis is still ongoing. Various hypotheses coexist. These hypotheses include the consumption of cannabis as a cause of psychosis, as a precipitating factor in certain individuals, or as a prolonger of symptoms, but they also encompass other factors that explain both phenomena or even psychosis as a cause of cannabis consumption [24]. The ethical impossibility of conducting experimental studies on this topic calls for caution when discussing etiological factors [25,26].

In addition to the intrinsic difficulties of a mental health disorder, individuals who suffer from it can also experience stigmatization. Stigma is a social construct that involves thoughts, emotions, and behaviors toward individuals diagnosed with a mental health disorder [27]. This stigma can be structural, personal (felt by the person themselves), and social stigma [28]. Despite having more information about mental health disorders owing to the emergence of communication technologies [29], there still exists effective discrimination in society, an intention of social distancing, and authoritarianism toward people who suffer from these disorders [30], partly due to beliefs of danger and incompetence regarding them [31]. It is important to emphasize the importance of seeking professional help for these individuals, providing support to their families, and developing anti-stigma campaigns [32,33,34]. Making proper causal attributions about what causes this stigma would aid in the promotion of these strategies. However, those with mental health disorders are not the only group affected by stigmatization. Other groups are also associated with stigmatization, either by others or even by themselves. Of all the possible groups, I will provide two additional examples. 

Stigma toward people with intellectual disabilities affects many aspects of their lives, including access to employment, housing, healthcare, and social care services. Furthermore, this stigma reduces their social opportunities, as they are perceived as “different” by society, and is even reflected in laws that diminish their autonomy [35]. People with intellectual disabilities are noted as one of the least preferred groups in social interactions, compared with other stigmatized groups [36]. Additionally, this group faces more negative attitudes than individuals with physical disabilities [37]. Interventions on the stigma of intellectual disability are scarce and not specifically targeted at stigma reduction [38,39]. Efforts to reduce stigma against individuals within these groups should encompass comprehensive interventions targeting attitudes, attributions, and behavioral intentions [38,40].

People experiencing homelessness also face significant stigmatization and are burdened by numerous negative stereotypes. They suffer structural stigma [41], which hinders their access to health services and resources [42]; personal stigma [43], where they internalize society’s prejudices and stereotypes, which in turn leads to social isolation [41]; and social stigma in the form of other’s negative perceptions and beliefs [44]. This collective faces social stigma primarily because homelessness is erroneously associated with personal traits such as laziness and antisocial behavior [41,45]. Unfortunately, these inadequate causal inferences obstruct the development of effective policies and prevention programs.

Understanding the factors that lead to stigmatizing attitudes toward these collectives (and others not specifically discussed here) is important for the implementation of programs, interventions, and other social policies. However, due to the correlational nature of studies investigating these issues, decisions must be cautiously made, supplementing empirical data with other theoretical criteria. Additionally, it is essential to continually review the changes brought about by interventions. The consequences of developing programs based on inadequate inferences are a wasteful expenditure of time and money and, of course, the program’s ineffectiveness on the well-being of individuals within the collective.

Another broad area where it is important to exercise caution when interpreting relationships between variables is that of addictive behaviors. I deliberately used the term “addictive behaviors” because I want to emphasize that we are dealing with a broad field that encompasses substance addictions but is not limited to them. I will only provide a brief overview of some examples within this field. 

There is societal concern about the abusive use of video games, associated with negative consequences such as sleep problems, discomfort, or the emergence of mental health or self-control issues [46]. The measurement of the extent to which video games can be a problem (beyond the diagnosis included in the DSM-V, [18]) is a subject of study [46,47]. For the prevention of and intervention in these problems, it would be advisable to know the etiological factors, but once again, this should be carried out with caution.

Gambling is a legal leisure activity in many countries. However, this activity can become pathological behavior. The factors associated with gambling problems include being male, irrational thoughts, high impulsivity, substance abuse, mental health issues, executive function deficits [48], accessibility and legislation, family factors, opinions or attitudes towards gambling [49], and advertising [50]. In other words, identifying the causes would be the key to prevention and treatment, as the determinants of pathological gambling may be found among a plethora of internal and external characteristics of the individual.

Substance abuse itself deserves an entire chapter. Abused drugs have been studied to understand the personal, social, environmental, or biological factors that lead to their consumption [51]. Understanding the variables related to vulnerability to substance abuse could help in developing interventions to reduce their consumption [52,53] or mitigate their most harmful effects [54]. 

Other examples of addictive behaviors can be found in workaholism, excessive physical exercise, and pornography consumption, among others. In all these health-related behaviors, causes should be studied with caution because, due to their nature, they cannot be subjected to experimental research.

## 5. Conclusions

This paper highlights the significance of methodology when interpreting empirical results, emphasizing areas of study that are highly relevant to people’s health. The fields of mental health, stigma, and addictive behaviors present multifaceted challenges that demand meticulous research methodology and careful interpretation of findings.

As in any other field where experimental methodology is impractical, unethical, or even illegal, the results are often derived within the framework of non-experimental designs. Generally, both editors and authors are aware that their conclusions cannot be stated as causal claims regarding the effect of one variable on another. However, it is still common to encounter studies that publish inappropriate, ambiguous, or erroneous causal expressions that can potentially confuse readers. By acknowledging the complexities and limitations of causal inference, we can contribute to more accurate and effective policies and interventions aimed at improving the well-being of individuals and society as a whole.
